# Modern Sociology

**Published:** 1899-03-18

**Authors:** 


### Modern Sociology.

### SEA-WATER FOR LONDON.

The water question is one which forces itself with ever-increasing weight on the attention of every municipality. How to obtain that "free gift of Heaven"—pure water—in sufficient quantity and at minimum cost has become everywhere the question of the age.

In spite of all the talk, statistics and capital expended on rival schemes by rival companies, the London water supply still threatens in the near future to become inadequate. The consumption of fresh water in London and district is calculated at not less than 200,000,000 gallons average a day, and of this about 20 per cent. is expended on mere domestic requirements. This difficulty may be met in various ways, among which may be mentioned the introduction of sea-water for municipal purposes.

Mr. Francis Newman, Civil Engineer, Borough Surveyor of Ryde, reports that so far back as 1854 "sea-water was used in Ryde for road watering," and that he "has recommended the Town Council to set up engine and pumps, and by pipes to convey sea-water to the upper part of the town that all the streets may be watered with it." As a result he reports that "it is found not only that one watering of sea-water is equal to two coats of fresh, but that it improves the road, making it harder and closer and less liable to break up."

The example of Ryde has since been followed by Great Yarmouth (1869), Blackpool, Bootle, Bournemouth, Barrow-in-Furness, Birkenhead, Falmouth, Fleetwood, Gosport, Grimsby, Harwich, Ilfracombe, Littlehampton, Londonderry, Plymouth, Portsmouth, Redcar, Shoreham, South Shields, Stockton-on-Tees, Torquay, Tynemouth, Weymouth, &c. In each case the substitution of sea-water for fresh was found to be in every way satisfactory. These experiments, to quote from a paper by Mr. Grierson, read before the Society of Arts, January 22nd, 1896, resulted in an Act of Parliament, passed 14 years ago, incorporating a company for the supply of sea-water to London. Satisfactory arrangements having been made with all the local authorities and other bodies affected, the Act was passed as an unopposed measure. The powers granted by that Act were, however, allowed to lapse in consequence of the great demands made by parishes, hotels, householders, and others for the supply of sea-water for public and private purposes. The directors were convinced that their plans had been laid on too small a scale, and that the authorised capital was insufficient to meet the requirements. The area in London to be dealt with now is much larger than that originally contemplated, and to meet its requirements a new company has been incorporated by Act of Parliament 1896. The London Sea Water Supply Company issues the following "Notes" on its prospectus: "This Act authorises the company to construct works to supply London with an unlimited quantity of sea-water; the pumps and settling tanks will be erected at Lancing, Sussex (where the sea is of unquestionable purity), to raise sea-water to an adjacent hill, whence it will flow by gravitation to London, passing through pipes laid in the public roads, and serving towns and places on the route. The mains will, by distributing pipes, supply the bath-rooms of private residents, hotels, hospitals, swimming baths, &c. Standpipes will be erected for watering the roads. Hydrants will also be placed for use in extinguishing fires. Sea-water is for these purposes more efficacious than fresh, and the City of London and many vestries have signified their desire to use it." This company is to have free play, and is unrestricted by Governmental interference. Its programme is briefly this: "To carry out a thoroughly practical and comprehensive scheme for the distribution of sea-water in London, which can, by additional works, be increased to meet any private or public demand, however great." Let us glance at this scheme, and note the four following points: (1) The intake; (2) pumping apparatus; (3) reservoirs; and (4) proposed main in London.

1. The place chosen for the intake is east of Lancing, between Brighton and Worthing.

2. Only one pumping station will be required, and is to be near the railway at Lancing, so that coal may be delivered from the trucks direct into the engine house. The water is to be pumped into a reservoir on the Downs, built on Seyning Hill, at an elevation of about 500 ft. The distance between the coast at Lancing and London is about 55 miles.

3. The present arrangement contemplates only two reservoirs besides the settling tanks near the sea, viz., that at Lancing and a large one for storage at Epsom. The fall between the two is about 260 ft.; the water, therefore, flows inland from the Downs by gravitation. The storage reservoir (at Epsom) is 240 ft. above the sea-level, an elevation which secures a pressure sufficient to force water flowing thence to London up to the tops of the highest buildings. In cases of conflagration the whole pressure from Lancing can be applied.

4. The main route in London will be viâ Battersea, Chelsea, Kensington, Paddington, Belgravia, &c., to Shoreditch, and will form a westerly and an easterly circuit. Service-pipes will be laid on from the main to public and private buildings, and to hydrants for the supply of water-carts. The popularity of sea-water for baths in hotels, hospitals, schools, asylums, and houses is likely to be considerable. It is said that the supply for municipal purposes will be at rates to compete with those now paid for fresh water.

